# Assessment model for multivariable fatigue performance of EA4T axles containing defects: ^a^*School of mechanical Engineering*,* Chengdu University*,* Chengdu 610,106*,* China*

**DOI:** 10.1038/s41598-025-34892-6

**Published:** 2026-01-08

**Authors:** Yan Luo, Gang Li, Chuanqi Qi, Cunhai Li, Yongxu Hu

**Affiliations:** 1 School of Mechanical Engineering, Chengdu University, Chengdu, 610106 China; 2 Sichuan Zichen Technology Co., Ltd., Chengdu, 611500 China; 3 Sichuan Aerospace Zhongtian Power Equipment Co., Ltd., Chengdu, 610100 China; 4 State Key Laboratory of Rail Transit Vehicle System, Southwest Jiaotong University, Chengdu, 610031 China

**Keywords:** EA4T axle, Surface defect, Full-scale axle, fatigue limit, Multivariate model, Engineering, Materials science, Mathematics and computing

## Abstract

This study employed a combined experimental and theoretical approach to investigate the influence of foreign object damage (FOD) on the fatigue limit of surface-strengthened EA4T axles. FOD was introduced on surface-strengthened axle specimens to generate surface defects, and finite element analysis was subsequently performed to evaluate the stress fields in the damaged regions. Fatigue tests were conducted on prefabricated defective specimens to characterize their fatigue behavior. Based on test results, an improved backward statistical inference method was used to fit the fatigue *P-S-N* curves for each specimen group and derive corresponding fatigue limits. The fatigue limit of full-size damaged axles was estimated by extrapolating from small-scale test results, with due consideration of geometric scale effects on mechanical performance. Considering the stochastic distribution of impact defect depths, an exponential fitting was performed to establish the quantitative relationship between defect depth and full-size axle fatigue limit. Finally, a multivariate fatigue limit prediction model was developed for surface-strengthened full-size EA4T axles based on the El-Haddad formula framework. This model enables comprehensive assessment of fatigue performance under multi-parameter coupling conditions, providing a robust theoretical basis for safety evaluation and maintenance strategies of high-speed train axles subjected to foreign object impacts.

**Supplementary Information:**

The online version contains supplementary material available at 10.1038/s41598-025-34892-6.

## Introduction

High-speed train axles are critical load-bearing components that support the entire weight of the train body^1,2^. During operation, extraneous objects such as track debris, small stones or airborne particles may impact the axle surface to induce foreign object damage (FOD). These disruptions not only compromise the geometric integrity of the axle but also cause stress concentrations and residual stress fields^3^. To tackle these challenges, surface enhancement technologies have emerged as effective strategies for extending fatigue life. One of the most promising options is the ultrasonic surface rolling process (USRP), which is characterized by high precision, flexibility, controllability and repeatability. Besides, it has been demonstrated remarkable capabilities in enhancing surface hardness, fatigue resistance and corrosion fatigue performance of metallic components^4–6^.

Over recent decades, extensive experimental and numerical investigations have been conducted to systematically evaluate FOD effects^7–9^. Statistical evidence indicates that approximately 30% of high-speed train axles have sustained foreign object impacts, highlighting the widespread occurrence of FOD and its significant implications for maintenance practices and operational safety in high-speed rail systems^10^. With the continuous increase in train speeds, researchers have increasingly turned their attention to investigating the issue of foreign object impact on high-speed train axles. Based on research into EA4T axles, Gao et al.^11^. demonstrated that the fatigue limit of indented specimens exhibits insensitivity to defect size. Additionally, the fatigue strength of specimens impacted by tungsten steel balls can likely be extrapolated from the relationship between the fatigue limit of indented specimens and indentation size.

The fundamental parameters in classical nominal stress and linear elastic fracture mechanics are fatigue strength and defects, respectively, and a quantitative relationship between the two can be established to some extent. In 1976, Japanese researchers Kitagawa and Takahashi proposed a novel concept that quantitatively links the threshold stress (fatigue limit) with the critical defect size, which is now known as the Kitagawa–Takahashi (K–T) diagram^12^. The introduction of the K–T diagram inspired further consideration of a possible quantitative correlation between the fatigue limit and the crack growth threshold. However, the original K–T diagram did not account for the short-crack effect. In light of this, using the El-Haddad model—which incorporates the short-crack effect—to modify the K–T diagram can yield a safer assessment in the near-threshold region^13^. Wu et al.^3,14^ systematically simulated FOD using a compressed‑gas gun and evaluated the fatigue strength and life of impacted EA4T axle specimens by applying a modified K‑T model within a fracture‑mechanics framework. This approach integrated experimental simulation of impact‑induced defects with theoretical modeling to characterize the relationship between defect morphology and fatigue performance. Similarly, by constructing a K-T diagram based on a single governing parameter for the S38C axle steel, it is demonstrated that the fatigue strength of impacted specimens lies within the region bounded by the characteristic curves of the diagram^15^. On the basis of the NASGRO and Chapetti models, the relationship between fatigue life and defect characteristics (size and location) was established by Hu et al.^16^, which not only allows for the estimation of fatigue life scatter but also supports the determination of a critical defect. Currently, the damage tolerance of railway axles serves as the foundational philosophy for their safe operation, encompassing defect analysis, residual lifetime prediction, and the reliability of inspection instruments. A thorough comprehension of FOD is indispensable for structural residual lifetime assessment and maintenance strategy formulation, both of which are critical for achieving a balance between safe operation and minimized maintenance costs^15^.

As previously mentioned, with the continuous increase in the operating speeds of high‑speed trains, the working conditions have become increasingly severe, and accidents arising from defects are progressively increasing. To ensure the safety, reliability, and cost‑effectiveness of equipment in service, it is of paramount importance to establish probabilistic *S–N* curves (*P‑S‑N* curves) based on representative material specimens^17^. Research indicates that through the collaborative probabilistic extrapolation method, the basic fatigue *P-S-N* curve can be jointly extrapolated into the very high cycle fatigue regime, thereby enabling the derivation of fatigue *P-S-N* curves for this life interval^18^. Xie^19^ introduced the backwards statistical inference method (BSIM), which establishes the relationship between standard deviation (STD) and applied stress to derive the converted fatigue life and achieve accurate probabilistic life prediction. To enhance the prediction accuracy for fatigue life data with limited sample sizes, an improved backward statistical inference approach (ISIA) was developed by incorporating modified distribution coefficients during the fitting of the *P-S-N* curve^20^.

However, existing research predominantly focuses on analyzing the influence of single variables on the fatigue performance of axle specimens. It is important to note that the fatigue strength of full-size axles differs inherently from specimen-level performance due to scale effects. Moreover, surface defects on axles typically arise randomly with uncertain defect depths. The random formation of defects and variations in defect depth create major uncertainties in fatigue performance. Thus, it is necessary to establish a multi-dimensional modeling for the evaluation of service performance of the axle. Therefore, this study conducted high-speed impact tests on surface-strengthened EA4T axle steel using foreign objects at varying velocities. Then, the finite element software was used to simulate the defect prefabrication process and characterize stress distribution at axle defect sites under different impact conditions. Fatigue tests on axle specimens yielded a high-survival-rate *P-S-N* curve and fatigue limit, which were then used to predict the full-size axle fatigue limit through scale effect correction. Finally, based on the El-Haddad formula, a three-dimensional fatigue performance evaluation model was established for full-size EA4T axles with impact defects to quantify the multi-parameter coupling influence on fatigue behavior. This approach combines experiments and modeling to provide a comprehensive framework for assessing axle fatigue reliability with complex impact-induced defects.

## Materials and methods

### Specimen preparation

The material used in this study was taken from the EA4T axle utilized in high-speed railway trains. Comprehensive analysis of the nominal chemical composition of EA4T steel is 0.27wt.% C, 0.39 wt.% Si, 0.72 wt.% Mn, 0.0075 wt.% P, 0.0013 wt.% S, 1.11 wt.% Cr, 0.01 wt.% Cu, 0.25 wt.% Ni, 0.25 wt.% Mo and balance Fe^21^.

**Fig. 1:**
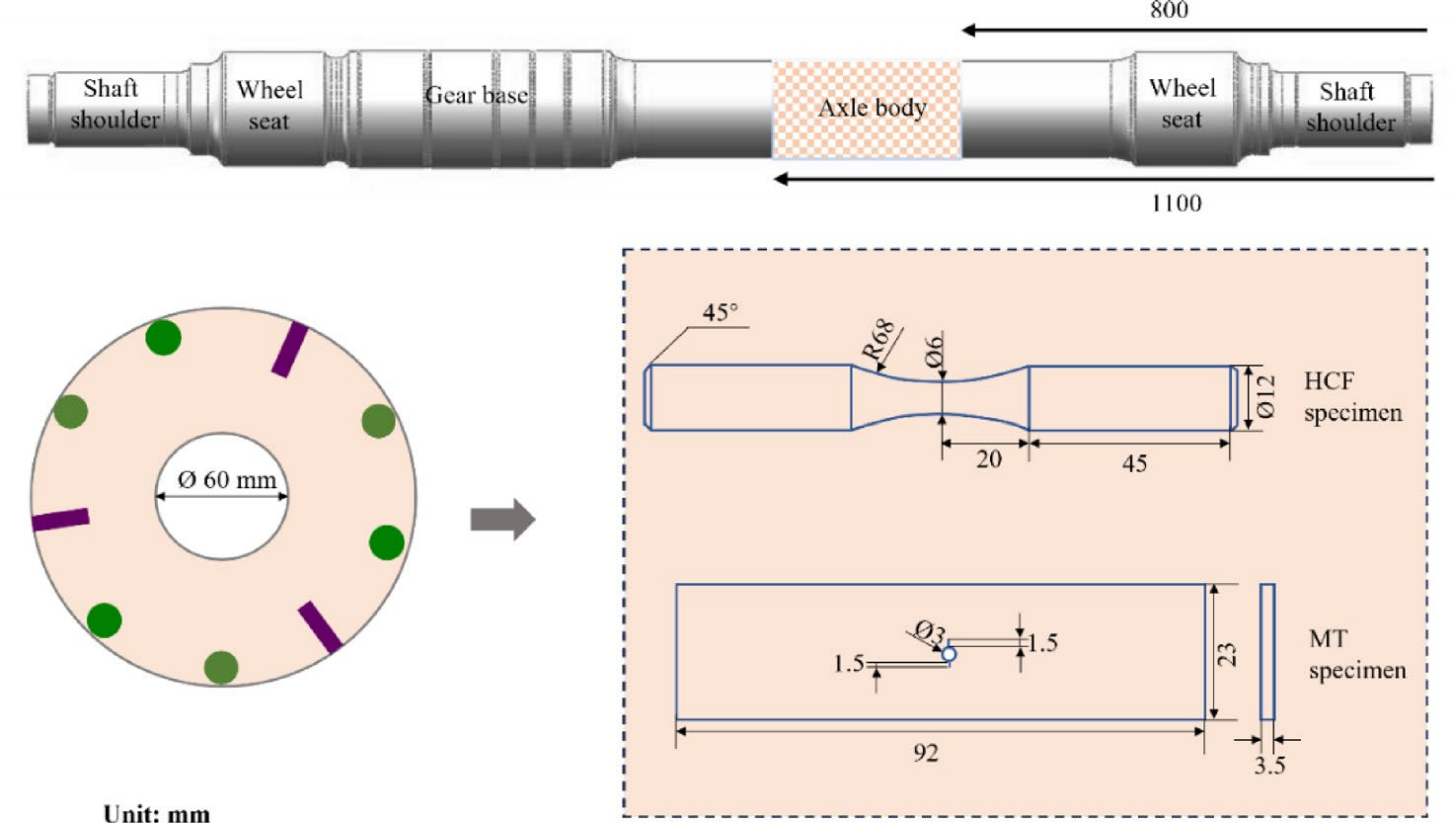
Sampling locations and dimensions of the specimens.

Specimens for fatigue testing, and fatigue crack growth (FCG) rate analysis were extracted from the EA4T axle body, a region frequently susceptible to foreign object damage (FOD). The precise sampling locations and their respective three-dimensional geometries are systematically depicted in Fig. 1

The HK30C ultrasonic rolling machine was utilized to conduct ultrasonic rolling treatment on the axle specimens. Previous research has demonstrated that the fatigue performance of the axle can be more effectively enhanced under the following optimal parameter set: a static pressure of 1085 N, two rolling passes, a feed rate of 0.12 mm/r, and a rotational speed of 50 r/min^21^.

### Simulation of FOD

To simulate FOD crater in a laboratory setting, a GCr15 steel ball with a diameter of 2 mm was propelled using a compressed gas gun facility at a velocity of 70, 100 and 167 m/s to induce surface impacts on the specimen at a 90° angle. The compressed gas gun, a high-speed ballistic impact apparatus was employed to replicate the targeted damage under controlled experimental conditions. The operational principle of the compressed gas gun device involves regulating the projectile launch velocity through controlled adjustments to the gas pressure within the high-pressure chamber. Projectile velocity is measured with a laser velocimetry system, and impact angle is calibrated using an adjustable fixture.

### High-cycle fatigue test

To investigate the fatigue strength characteristics, rotary bending high-cycle fatigue (HCF) tests were systematically performed on both smoothed and surface-damaged hourglass-shaped specimens under ambient atmospheric conditions. The experimental protocol employed a QBWP–6000 J rotary bending fatigue testing apparatus, with cyclic loading frequency set within the 34–36 Hz range (mechanically equivalent to the operational velocity of 360 km/h for high-speed electric multiple unit (EMU) trains) under a stress ratio of *R* =  − 1. Prior to testing, all specimens underwent standardized surface preparation procedures involving sequential grinding and polishing operations, yielding controlled surface roughness parameters (*R*_a_) within the range of 0.6–0.8 μm.

### Fatigue crack growth rate test

The fatigue crack growth (FCG) behavior was experimentally characterized utilizing an Amsler-HFP5000 high-frequency servo-hydraulic testing system. Middle-crack tension (MT) specimens, machined from the as-received axle body material (geometric details illustrated in Fig. 1), were subjected to cyclic loading under displacement-controlled conditions. A pre-crack with a length of 1.5 mm was intentionally introduced at the central bore of the specimen. The sinusoidal waveform loading was applied at a frequency range of 120–150 Hz with fully reversed stress ratio (*R* = − 1)^3,22^. To establish the threshold stress intensity factor range (Δ*K*_th_), a standardized *K*-decreasing methodology was implemented. Subsequently, the FCG rate (d*a*/d*N*) versus Δ*K* relationship was quantified through a *K*-increasing loading regimen. The crack length was determined by the compliance method during propagation.

### Finite element modeling

The accuracy of the impact simulation depends on selecting appropriate material constitutive models. After evaluating the theoretical framework and validation data, the Johnson–Cook (J-C) model was chosen to describe velocity impact dynamics. The governing equations are^23^:

1 $$\sigma = \left( {A + B\varepsilon_{{\mathrm{p}}}^{n} } \right)\left[ {1 + C\ln \left( {\frac{{\mathop {\varepsilon_{{\mathrm{r}}} }\limits^{.} }}{{\mathop {\varepsilon_{{{\mathrm{r}}0}} }\limits^{.} }}} \right)} \right]\left[ {1 - \left( {\frac{{T - T_{0} }}{{T_{{\mathrm{m}}} - T_{0} }}} \right)^{m} } \right]$$

where *σ* is the yield stress, *A* is the yield strength, *B* is the hardening modulus, *n* is the hardening index, *C* is the strain rate hardening parameter, *m* is the thermal softening coefficient, *ε*_p_ is the plastic strain, *ε*_r_ is the strain rate and *ε*_r0_ is the reference strain rate.

Based on the research results of Zhao Xin et al.^23^, the fitting parameters *A*, *B*, *n*, and *C* of the J-C constitutive equation of EA4T axle steel at room temperature were obtained as *A* = 589 MPa, *B* = 358 MPa, *n* = 0.27, and *C* = 0.018, respectively.

**Fig. 2:**
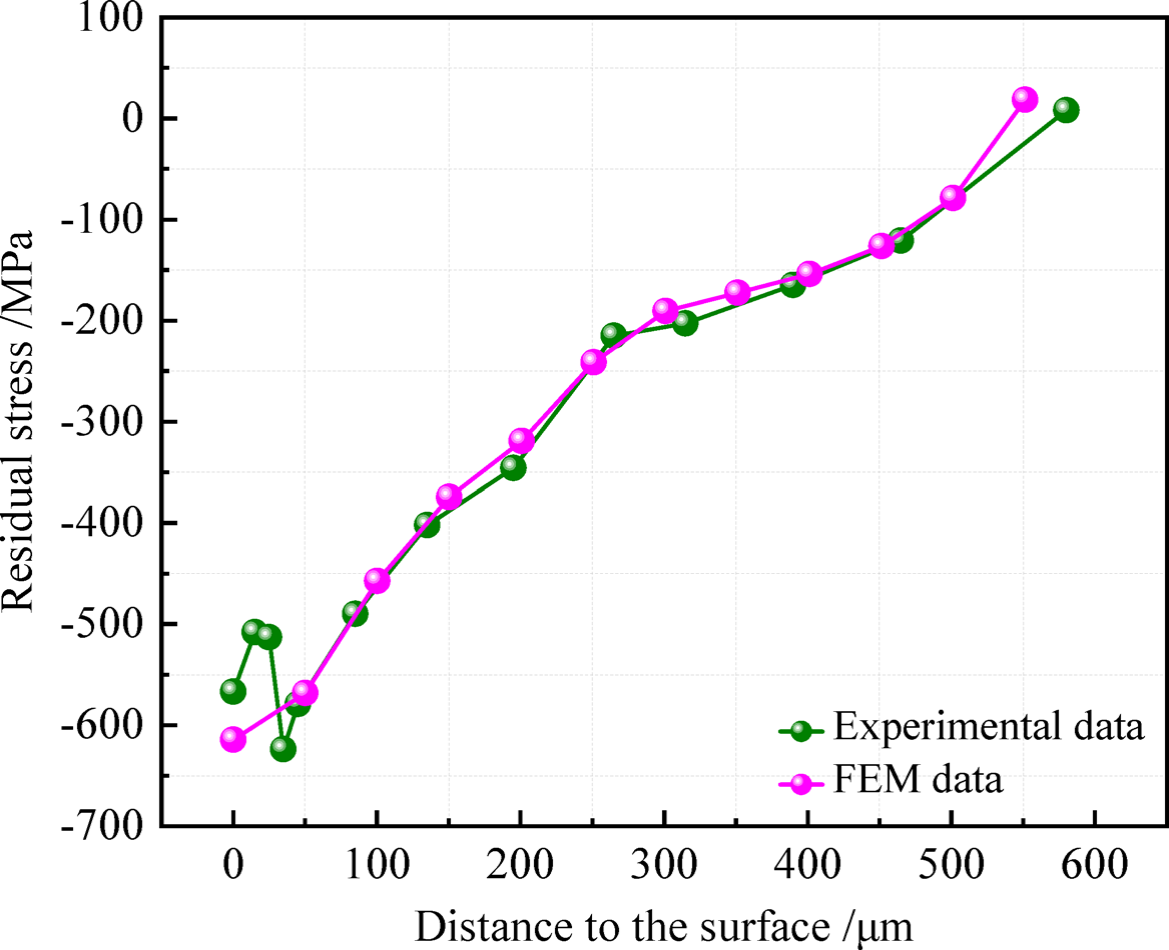
Comparison of the FEM values of the residual stress with the test data.

USRP imparted a pronounced residual compressive stress field within the surface and subsurface layers of components. Accordingly, the accurate representation of this stress field is critical in finite element simulations. This study introduced temperature field modulation simulation strategy in which a compressive stress field, analogous to that induced by USRP, was generated within the strengthened layer of an axle specimen^24,25^. The targeted compressive stress field within the strengthened layer was subsequently facilitated through the controlled application of these thermal gradients. The residual stress distribution within the strengthened layer was first determined experimentally via a hybrid analytical approach combining sequential electrolytic layer removal with non-destructive X-ray diffraction (XRD) stress analysis, as depicted in Fig. 2. A corresponding residual compressive stress field was then established through numerical simulation. The gradient distribution of the simulated residual stress was extracted and validated against the experimental measurements (Fig. 2). The results demonstrate favorable agreement between the simulated and experimental data.

**Fig. 3:**
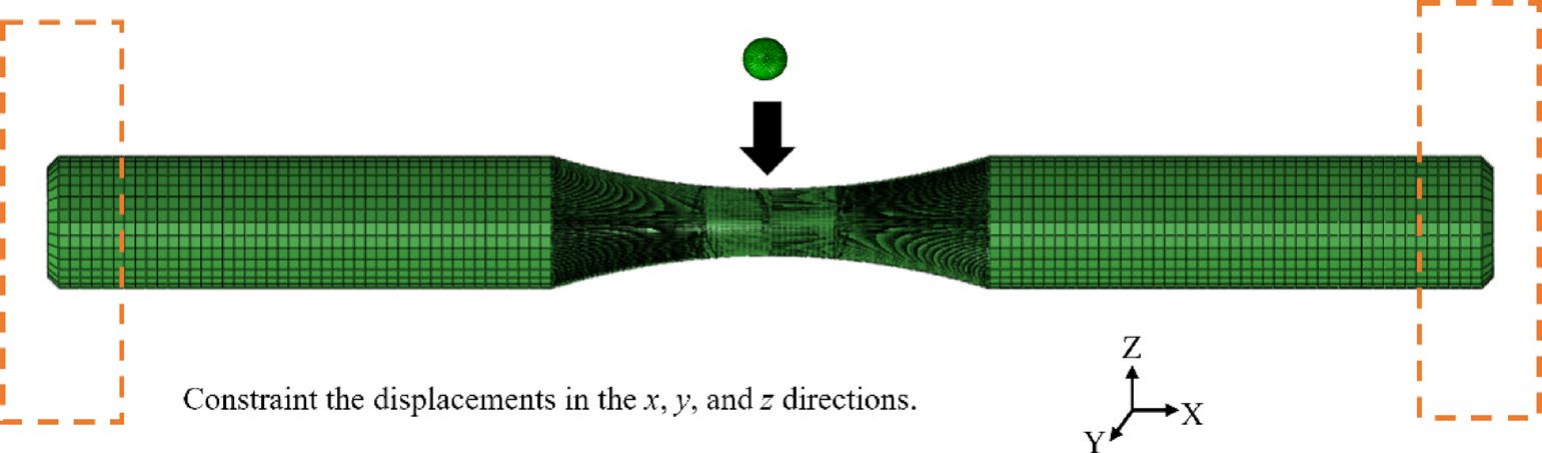
Finite element model of the impact process.

In the course of impact simulation computations, spherical projectiles with a diameter of 2 mm were employed, and impact velocities of 70 m/s, 100 m/s, and 167 m/s were respectively imposed. The impact process was simulated using the ABAQUS/Explicit finite element code, with the target specimen discretized by eight-node reduced-integration hexahedral (C3D8R) elements. Following a mesh sensitivity study, the element size in the impact region was refined to 0.025 mm to ensure accuracy^23^. In other regions, a coarser mesh was adopted to optimize computational efficiency without compromising the validity of the results. The final finite element model comprised 26,149,425 elements and 26,383,202 nodes. The displacements in the* x*, *y*, and *z* directions were constrained at both ends of the specimen. The foreign object was launched at initial velocities of 70 m/s, 100 m/s, and 167 m/s, consistent with the FOD simulation tests, as shown in Fig. 3.

## Results

### Foreign object damage characterization

**Fig. 4:**
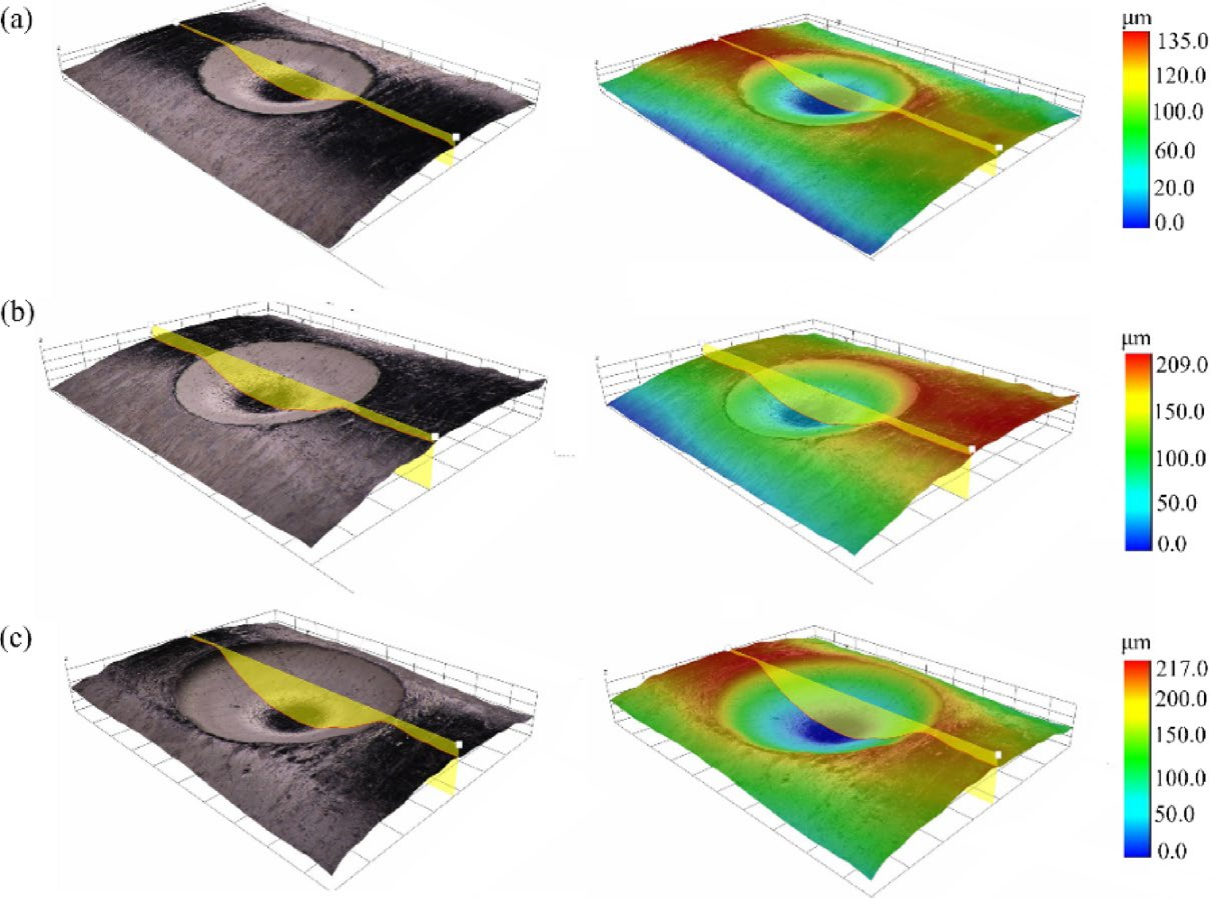
Foreign object impact crater, (**a**)* v* = 70 m/s, (**b**) *v* = 100 m/s, (**c**) *v* = 167 m/s.

Figure 4 presents the surface profiles and three—dimensional (3D) profiles of the specimens with three different defect depths that were experimentally investigated. The overall morphological characteristics of these defects bear a resemblance to a crater. A positive correlation is observed between impact velocity and subsurface deformation severity, where increasing impact energy induces progressively intensified plastic deformation at the crater base, accompanied by corresponding augmentation in defect depth. More precisely, the average depths* d* of these defects are measured as 72 μm when the impact velocity *v* = 70 m/s, 110 μm at *v* = 100 m/s, and 210 μm for* v* = 167 m/s.

### Stress distribution characterization

**Fig. 5:**
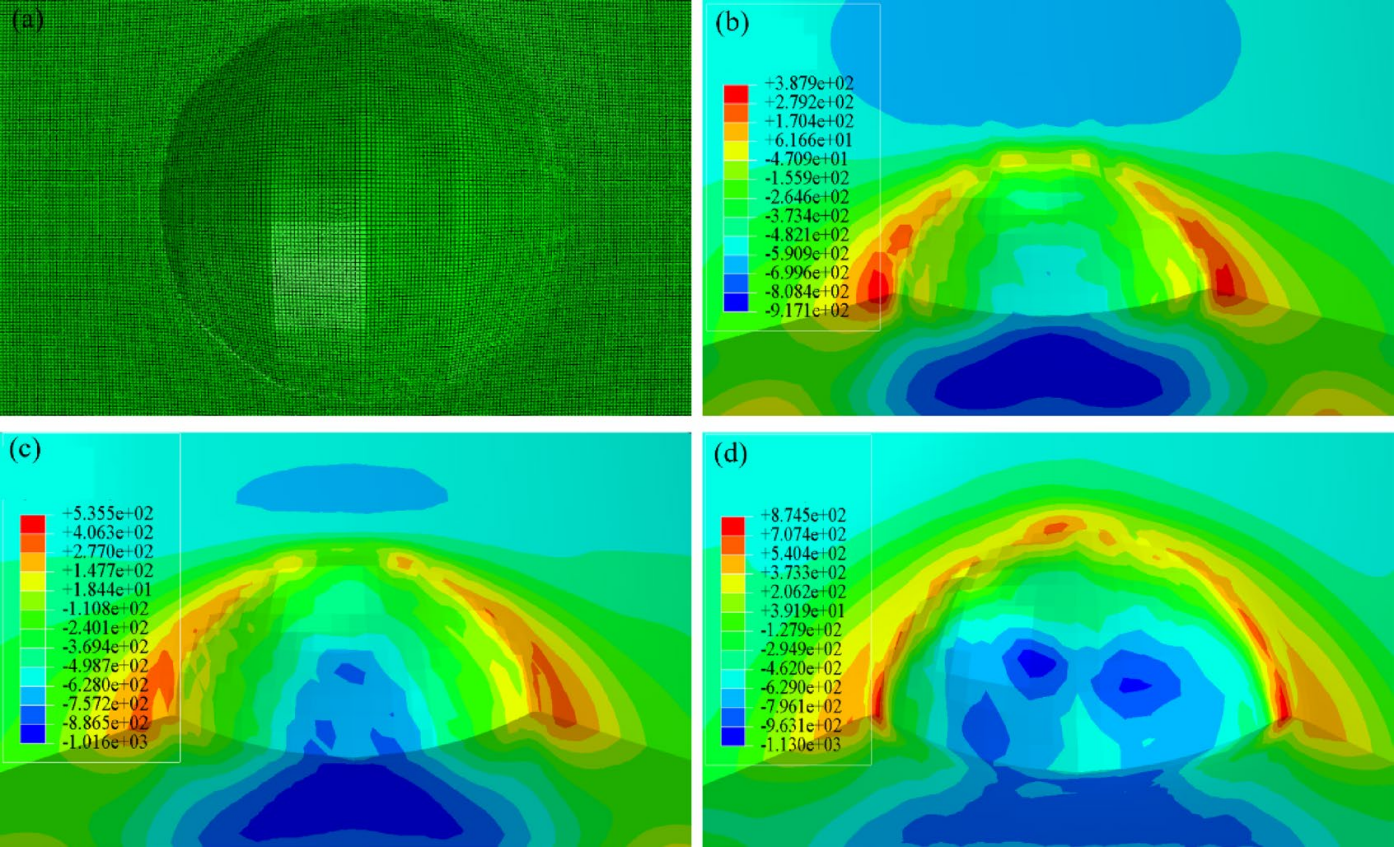
Stress distribution at impact crater, (**a**) foreign object impact crater, (**b**)* v* = 70 m/s, (**c**) *v* = 100 m/s, (**d**) *v* = 167 m/s.

During operation, rotating bending loads induce critical stress components along the axle’s axial direction (denoted as *S*_33_), which predominantly govern fatigue crack initiation and propagation^26,27^. Figure 5 presents the* S*_33_ stress distribution from finite element analysis. The results reveal a complex spatial stress pattern with high gradients after foreign object impact. High tensile stresses concentrate around defect edges, while compressive stresses are mainly in the defect bottom regions. Foreign object impact induces pronounced surface plastic deformation in specimens, generating a complex residual stress field. Subsequent external loading triggers stress concentration within these deformed zones, ultimately leading to crack initiation at defect sites.

### High cycle fatigue properties

The fatigue *S–N* characteristics of railway axle steel specimens under various surface damage conditions are illustrated graphically in Fig. 7.

The Basquin equation is empirically formulated as follows^28,29^:

2 $$S_{a} = \sigma_{f}{\prime} \left( {2N_{f} } \right)^{b}$$

where *S*_*a*_ denotes the stress amplitude, *σ*′_*f*_, the fatigue strength coefficient, *N*_*f*_, the number of cycles to failure; and *b*, the fatigue strength exponent.

Accordingly, *σ*′_*f*_ and *b* for the smoothed, FODed-*v* = 70 m/s, FODed-*v* = 100 m/s and FODed-*v* = 167 m/s specimen can be determined from Fig. 7., as listed in Table 1. The data in the table show that fatigue strength exponent *b* exhibits a decreasing trend with increasing impact speed. This behavior can likely be attributed to stress concentration arising from the defect^30^.

**Table 1:** Fatigue property parameters of different specimens.

Specimen	Fatigue strength coefficient *σ*′_*f*_	Fatigue strength exponent *b*
Smoothed	1176	− 0.0631
FODed-*v* = 70 m/s	1420	− 0.0826
FODed-*v* = 100 m/s	1727	− 0.1066
FODed-*v* = 167 m/s	1730	− 0.1137

To ensure robust and conservative characterization of fatigue strength, a probabilistic *P-S-N* curve with 97.5% confidence level was constructed through the ISIA^20^. Assuming that the logarithm of fatigue life follows a normal distribution across all stress levels, the consistency of percentiles can be expressed as^19^:

3 $$\frac{{\log N_{ji} - \mu_{j} }}{{\sigma_{j} }} = \frac{{\log N_{ki} - \mu_{k} }}{{\sigma_{k} }}$$

where *μ*_*j*_ and *σ*_j_ are the mean value and standard deviation, respectively, of the log-transformed fatigue life log *N*_ji_ at stress level *j*.

**Fig. 6:**
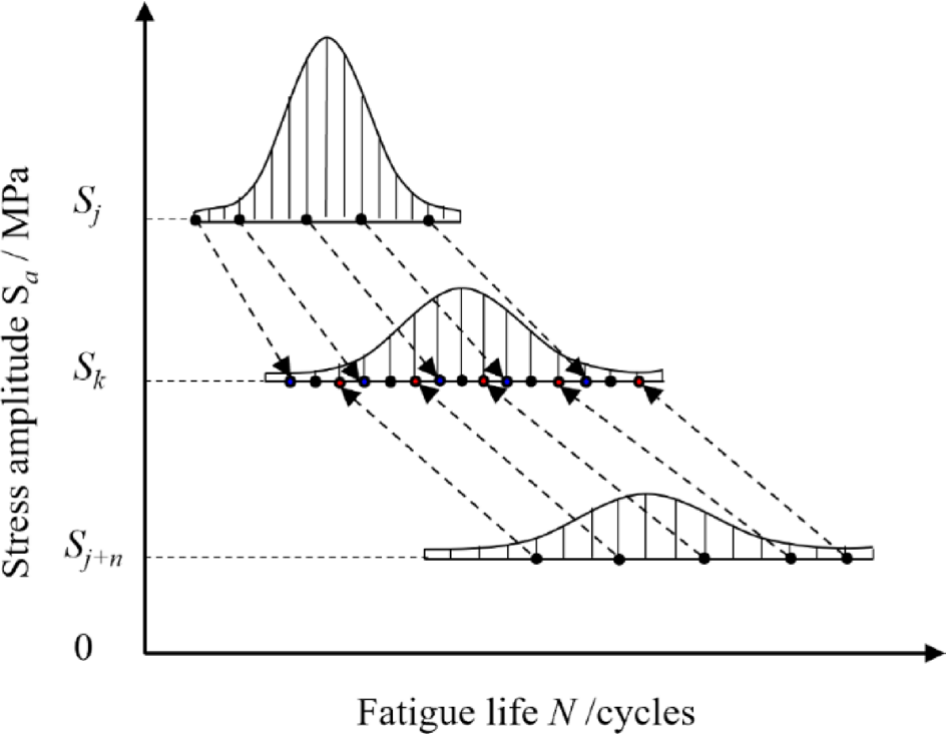
Schematic of fatigue life equivalence across stress levels^20^.

**Fig. 7:**
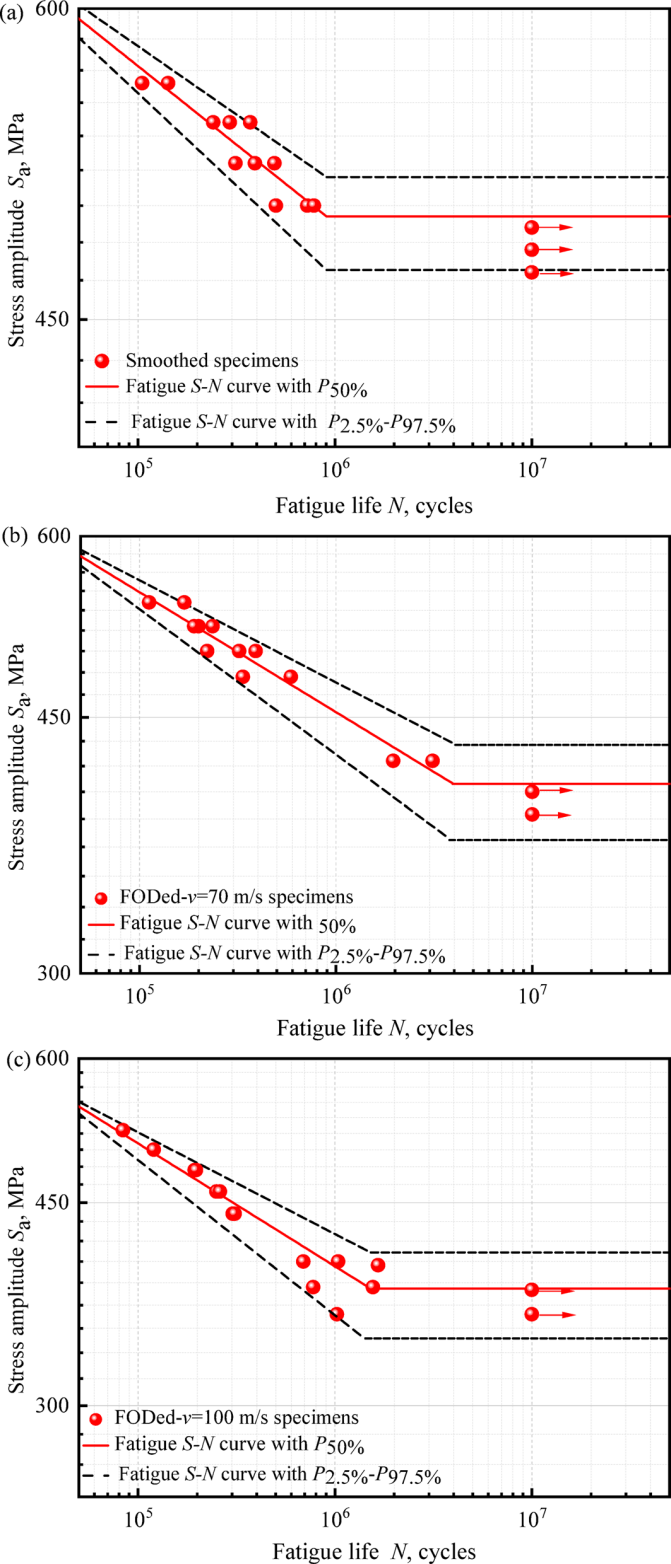

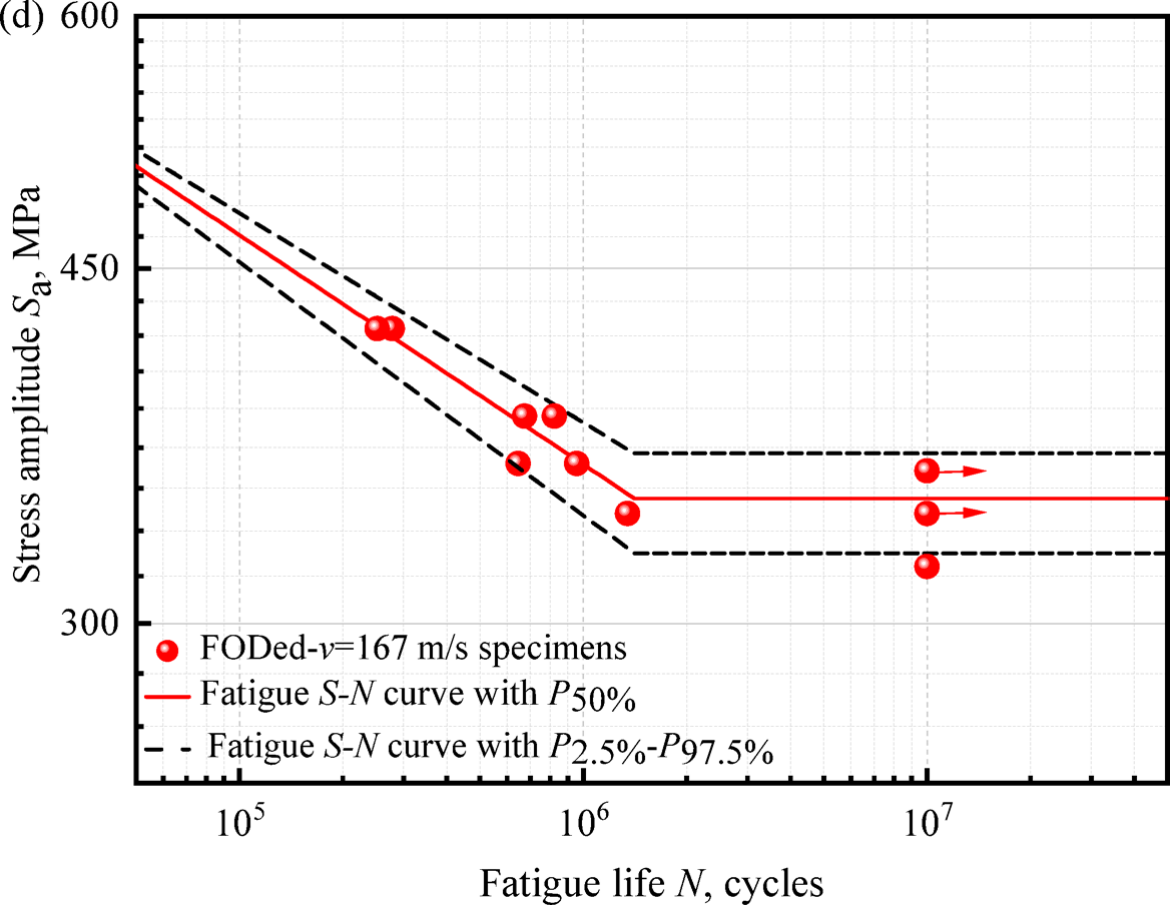
*S–N* curves for EA4T axle specimen: (**a**) smoothed, (**b**) FODed-*v* = 70 m/s, (**c**) FODed-*v* = 100 m/s, (d) FODed-*v* = 167 m/s.

Based on the equivalence established in Figs. 6 and 7, the fatigue life at stress level *k* can be formulated in terms of its counterpart at stress level *j.*

4 $$\log N_{ji}^{e} = \left( {\log N_{ji} - \mu_{j} } \right)\frac{{\sigma_{k} }}{{\sigma_{j} }} + \mu_{k}$$

where log *N*_*ji*_^e^ denotes the equivalent value of log *N*_*ji*_ at stress level *k.*

The mean fatigue life at each stress level is derived from the median *S–N* curve, while the standard deviation is determined as follows^19,31^:

5 $$\sigma_{i} = \sigma_{k} + K\left( {S_{k} - S_{i} } \right)$$

Therefore, the standard deviation *σ*_*k*_ at stress level *k* serves as the basis for estimating the standard deviations at all other stress levels.

Table 2 and Table 3 list the standard deviations for each specimen across the tested stress levels, as derived from Eqs. (4) and (5) ^20^.

**Table 2:** The fatigue life distribution parameters of smoothed and FOD-*v* = 70 m/s specimens.

Smooth			FOD-*v* = 70 m/s			
Stress level/MPa	Mean value	STD		Stress level/MPa	Mean value	STD
560	5.11	0.0817		540	5.09	0.0651
540	5.36	0.1015		520	5.28	0.0849
520	5.62	0.1213		500	5.49	0.1047
500	5.89	0.1411		480	5.71	0.1245
				420	6.41	0.1839

**Table 3:** The fatigue life distribution parameters of FOD-*v* = 100 m/s and FOD-*v* = 167 m/s specimens.

FOD-*v* = 100 m/s			FOD-v = 167 m/s			
Stress level/MPa	Mean value	STD		Stress level/MPa	Mean value	STD
500	5.05	0.0581		420	5.41	0.0735
480	5.22	0.0779		380	5.79	0.0931
460	5.39	0.0977		360	5.99	0.1029
440	5.57	0.1175		340	6.21	0.1128
400	5.96	0.1571		420	6.41	0.1839
380	6.17	0.1769				

Consistent with fatigue mechanics principles, the cyclic durability of EA4T axle steel exhibits a statistically significant inverse correlation with applied stress amplitude under controlled loading conditions. The experimentally determined fatigue limits demonstrate a progressive degradation from 495 MPa (smoothed specimen, STD = 21.07) to 346 MPa with increasing impact velocities: (i) 70 m/s (405 MPa, STD = 30.09), (ii) 120 m/s (379 MPa, STD = 32.08), and (iii) 167 m/s (346 MPa, STD = 19.52). As illustrated in the figures, the fatigue resistance of the specimens exhibits a monotonic reduction inversely proportional to surface flaw depth progression under sustained loading conditions.

Furthermore, the fatigue limits from the 97.5% reliability *P-S-N* curve are: 471 MPa for the smoothed specimen, 370 MPa at *v* = 70 m/s, 343 MPa at *v* = 100 m/s, and 325 MPa at *v* = 167 m/s. These results show the *P-S-N* curve method improves safety and reliability assessment of axle components.

### Fatigue fracture analysis

**Fig. 8:**
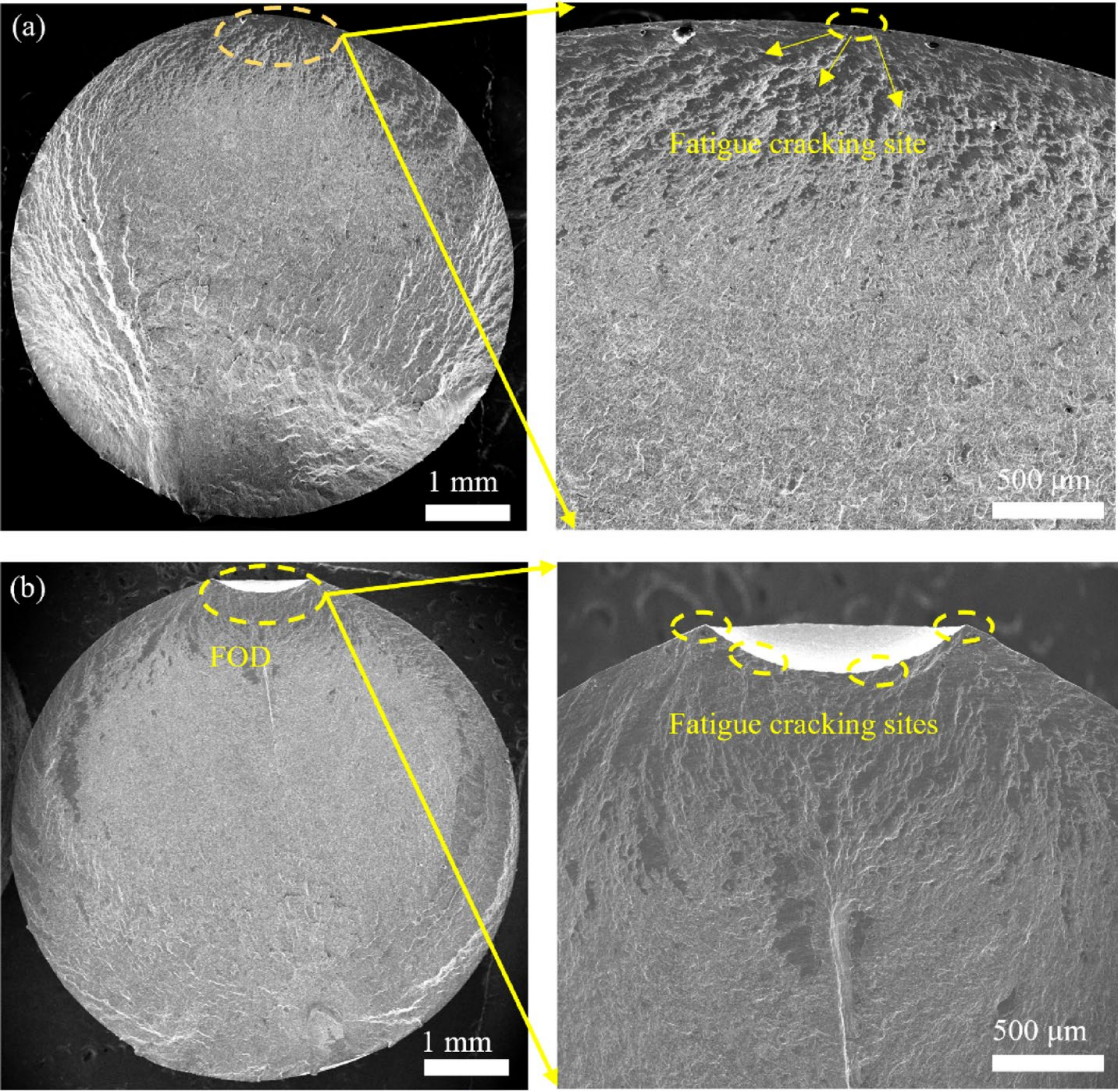
Fracture characteristics of the surface strengthened EA4T axle specimens: (**a**) macroscale view of a smoothed specimen (fatigue life of 1,713,680 cycles), (**b**) macroscale view of a FODed specimen at *v* = 167 m/s (fatigue life of 823,017 cycles).

Figure 8 presents comparative analysis of fatigue fracture morphologies between smoothed and FODed specimens. As illustrated in Fig. 8a, macroscopic examination reveals that fatigue cracks in the smoothed specimen predominantly initiate at the surface region. However, in FODed specimens, crack initiation predominantly occurs circumjacent to the defect sites. The FOD-induced impact pits structurally resemble surface micro-notches, generating characteristic stress concentrations quantified by the theoretical stress concentration factor, *K*_t_ = 1 + 2√(*d*/*r*), which promote the fatigue crack to initiate. Besides, the presence of FOD-induced defects fundamentally alters the stress field distribution characteristics of the original specimen^32^. This stress redistribution mechanism establishes defect zones as critical nucleation sites for fatigue crack initiation, as evidenced by fracture surface analysis (see Fig. 8b).

### Fatigue crack growth properties

Fatigue crack propagation behavior critically governs the fatigue-resistant design of component^33^. This necessitates systematic investigation of threshold stress intensity factor range (Δ*K*_th_) and crack growth rate (*d*a/*d*N) in surface-damaged specimens with USRP treatment, to establish reliable damage tolerance assessment criteria^3,34^.

**Fig. 9:**
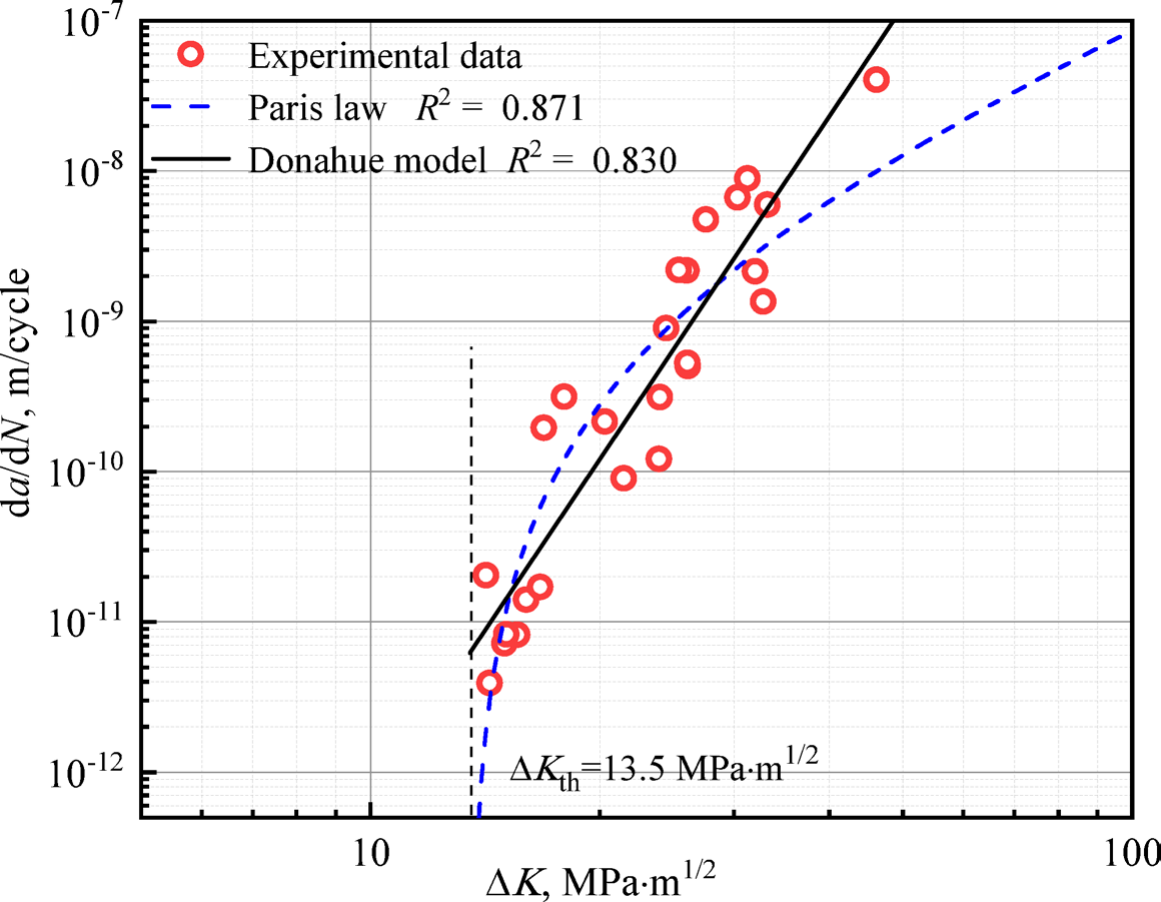
Fatigue crack growth rate of USRPed axle steel and fitted Paris/Donahue models.

Figure 9 demonstrates the crack growth rate curves of surface-strengthened axle specimens. To date, a multitude of models have been formulated in the literature to characterize the FCG behavior^35^. In the present study, the well—established Paris law^36^ and the Donahue model^37^ are utilized to model the fatigue crack growth rate curve.

The foundational Paris formula, which models crack growth rate, was proposed by Paris and Erdogan^36^ and is given in Eq. (6). This empirically derived model correlates the crack growth rate (d*a*/d*N*) log-linear with the stress intensity factor range (∆*K*). Owing to its effectiveness in capturing the fundamental mechanics of crack propagation, it has become a cornerstone of fracture mechanics:

6 $$\frac{{{\mathrm{d}}a}}{{{\mathrm{d}}N}} = C(\Delta K)^{m}$$

where d*a*/d*N* is the FCG rate, Δ*K* is the stress intensity factor range, *C* and *m* are the material’s constants (here fitted as 1.770 × 10^–20^ and 7.561, respectively).

Since the Paris model does not account for the characteristics of the threshold regime, Donahue et al.^37^ introduced a crack growth threshold parameter to modify the conventional Paris model:

7 $$\frac{{{\mathrm{d}}a}}{{{\mathrm{d}}N}} = A(\Delta K - \Delta K_{{{\mathrm{th}}}} )^{n}$$

where* A* and *n* are the material’s constants (here fitted as 4.395 × 10^–12^ and 2.216, respectively).

As shown in the Fig. 9, in comparison to the Paris model, the Donahue model provides a superior delineation of the threshold regime characteristics. Furthermore, it also better captures the crack growth behavior of surface-strengthened axle specimens across the available dataset.

## Discussion

### Fatigue limit prediction for full-scale railway axles

Despite the fact that the axle specimen and the full-scale axle are fabricated from identical materials, pronounced discrepancies exist in their geometric dimensions. Additionally, disparities are evident in the material surface processing degree and the applied loading conditions. These multi-faceted factors collectively exert a substantial influence on the fatigue limit of the components. Consequently, the combined effect of these factors necessitates careful consideration when extrapolating fatigue performance data from axle samples to full-scale axles^38,39^. To establish a systematic framework for predicting fatigue limits in defect-containing high-speed railway axles, rigorous characterization of geometric scaling effects (specimen-to-component transferability) and surface integrity parameters is prerequisite. The experimentally determined fatigue limits for FOD-impacted specimens at 97.5% reliability levels in the probabilistic *P-S-N* curves require correction through validated transfer functions to enable reliable full-scale axle damage tolerance assessment^40^:

8 $$\sigma_{{\mathrm{a,fs}}} = \sigma_{{\mathrm{a,lf}}} \alpha \beta \varepsilon$$

where, *σ*_a,fs_ is the predicted fatigue limit of the full-scale axle, *σ*_a,ls_ represents the fatigue limit of the specimen, *α* is the load coefficient (*α* = 1), *β* is the surface quality coefficient (*β* = 0.9), *ε* is the geometry coefficient (*ε* = 0.86)^40^*.*

**Fig. 10:**
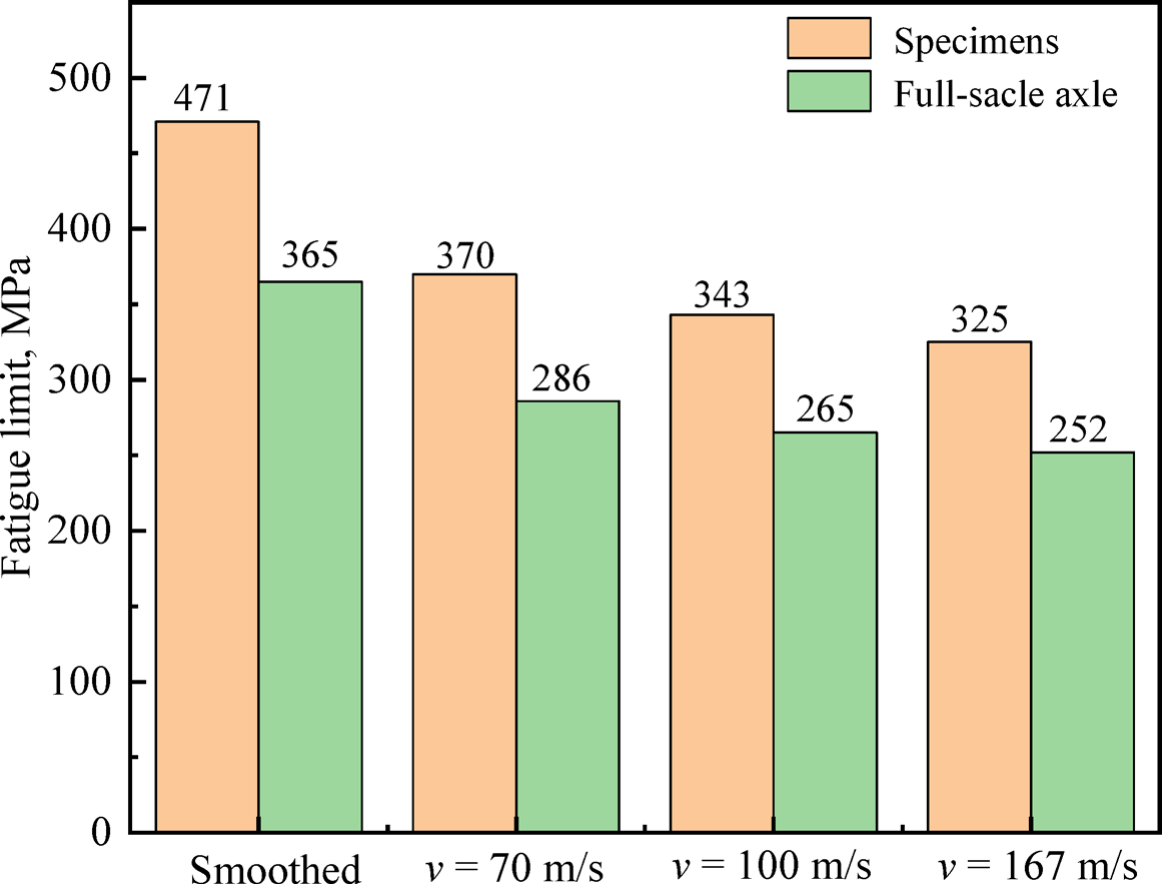
Fatigue limit for full-scale axle and specimens.

The predicted fatigue limits of full-scale railway axles for each specimen group, derived from Eq. (8), are compared with the corresponding specimen-level fatigue limits in Fig. 10. The predicted fatigue limits for full-scale axles are consistently lower than the experimentally measured values of their corresponding small specimens. Specifically, the smoothed full-scale axle shows a deficit of 106 MPa, while the FODed axles exhibit deficits of 84 MPa, 78 MPa, and 73 MPa at impact velocities of 70 m/s, 100 m/s, and 167 m/s, respectively. Consequently, when assessing the safe fatigue-loading region of an axle, full consideration of the detrimental effects caused by differences in geometry, surface quality, and loading method between test specimens and the actual axle can result in more reasonable and conservative evaluation outcomes.

**Fig. 11:**
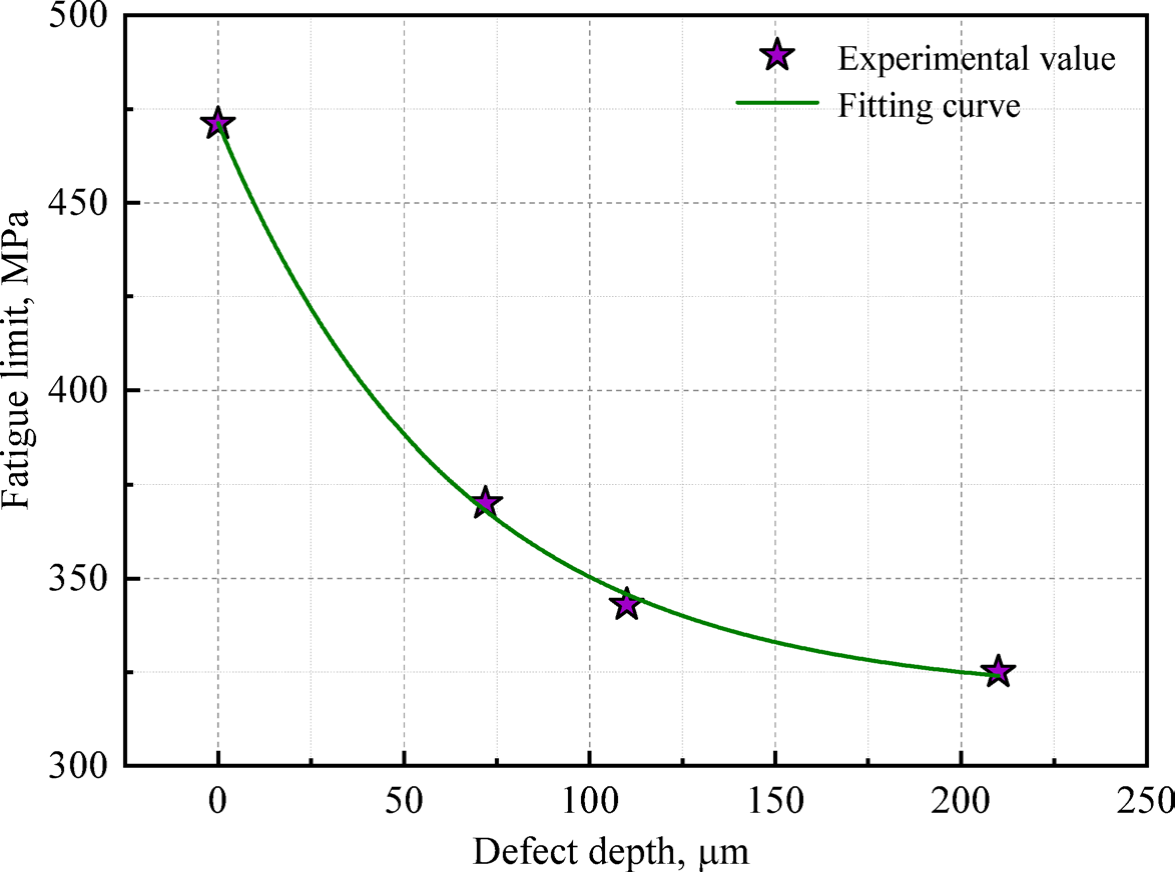
Dependence of fatigue limits on FOD-induced defect depths in full-scale axles.

Experimental results establish a quantifiable relationship between FOD-induced defect depth and subsequent fatigue limit. The studies demonstrated that notch size effects on fatigue characteristics follow an exponential decay pattern, with mathematical modeling confirming the inverse correlation between defect dimensions and material endurance limits, as shown in Eq. (9) ^41–43^. Figure 11 presents the mathematical relationship between fatigue limits and defect depths in FOD-affected specimens through regression analysis. The statistical modeling reveals a negative exponential dependence of fatigue limits on defect depth. Equation (10) analytically formulates the functional dependence of fatigue limits on defect depths under FOD-impacted conditions.

9 $${\mathrm{y}} = y_{0} + A_{0} e^{{(x - x_{0} )/t_{0} }}$$

10 $$\begin{gathered} \sigma_{{{\mathrm{w0}}}} = 318 + 153e^{( - d/64)} \hfill \\ d \le 210{\mu m} \hfill \\ \end{gathered}$$

where, *σ*_w0_ is the full-scale axle fatigue limit at *R* = -1, *d* is the defect depth.

### Reliability-based quantification for surface defect criticality in full-scale axle

As a benchmark methodology for assessing defect tolerance in materials and structural components, the K-T diagram integrates fatigue strength concepts with fracture mechanics principles through a semi-empirical framework, providing a graphical representation of fatigue limit dependence on defect size^44–46^. While the conventional K-T diagram overlooks short crack behavior and near-threshold regime effects in fatigue strength evaluation of defective materials and structural components, its modified iteration incorporates El Haddad’s intrinsic crack length concept to address these limitations^13^:

11 $$\Delta K_{{\mathrm{th,L}}} = F_{{\mathrm{w}}} \cdot \Delta \sigma_{{{\mathrm{th}}}} \cdot \sqrt {{\uppi }a}$$

12 $$\Delta \sigma_{{{\mathrm{th}}}} = \frac{{\Delta K_{{\mathrm{th,L}}} }}{{F_{{\mathrm{w}}} \cdot \sqrt {{\uppi }a} }}$$

13 $$\Delta K_{{\mathrm{th,L}}} = F_{{\mathrm{w}}} \cdot \Delta \sigma_{{{\mathrm{th}}}} \cdot \sqrt {{\uppi }\left( {a + a_{0} } \right)}$$

14 $$\Delta \sigma_{{{\mathrm{th}}}} = \frac{{\Delta K_{{\mathrm{th,L}}} }}{{F_{{\mathrm{w}}} \cdot \sqrt {{\uppi }\left( {a + a_{0} } \right)} }}$$

15 $$a_{{0}} = \frac{1}{{\uppi }}\left( {\frac{{\Delta K_{{{\mathrm{th}}}} }}{{F_{{_{{\mathrm{W}}} }} \Delta \sigma_{{{\mathrm{w0}}}} }}} \right)^{2}$$

where, Δ*σ*_th_ is the fatigue threshold stress range, Δ*K*_th, L_ is the long fatigue crack propagation threshold (here, Δ*K*_th, L_ = 13.5 MPa⋅m^1/2^), *F*_W_ is the geometric factor (here *F*_W_ = 0.65 for the surface defects), *a* is the crack length, *a*_0_ represents the modified El-Haddad parameter.

**Fig. 12:**
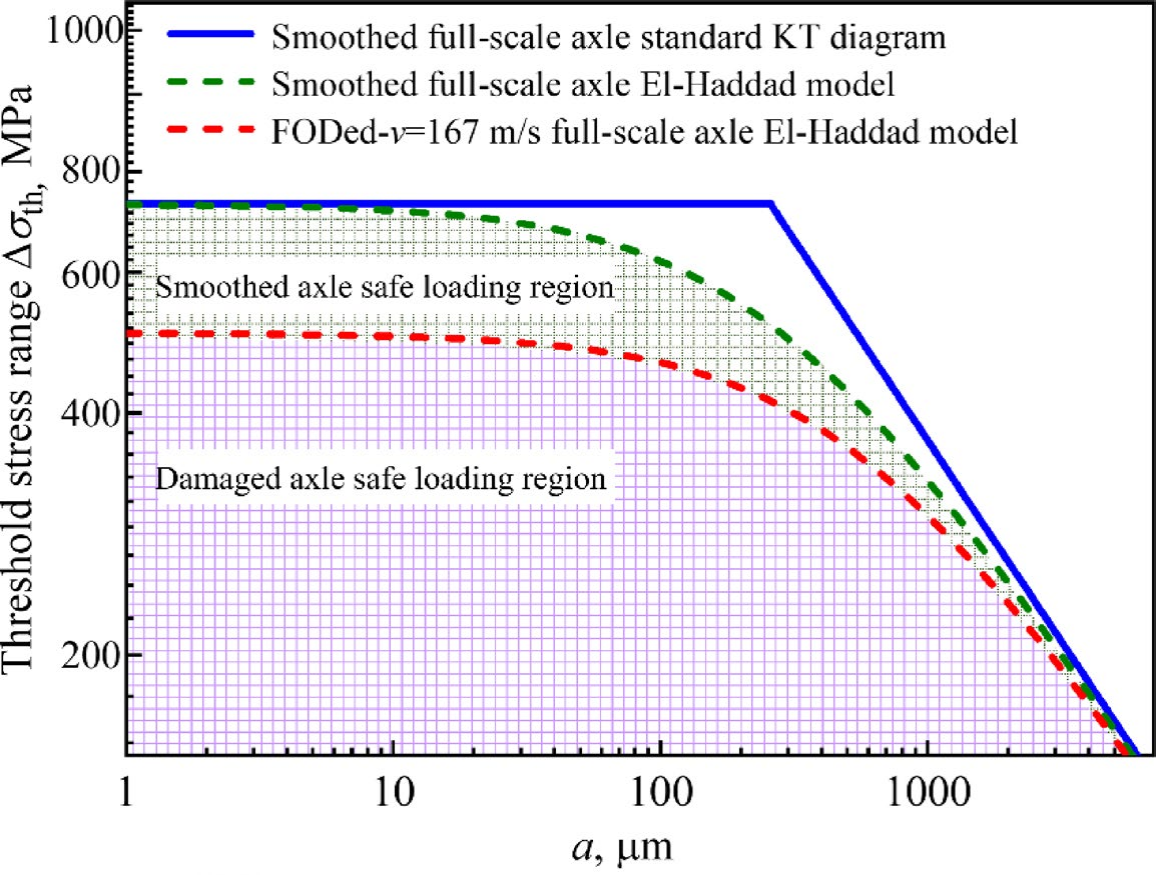
Full-scale damaged axle modified K-T diagram.

Consequently, utilizing the fatigue limit derived from full-scale axles with 97.5% reliability and the long crack propagation threshold, the intrinsic crack length (*a*₀) is determined as 258 µm. Both the standard and modified K-T diagrams are subsequently constructed for smoothed and FODed (*v* = 167 m/s) axles, as illustrated in Fig. 12. The region beneath the curve defines the safe loading zone. In contrast to the standard K-T diagram, the modified diagram for damaged axles exhibits a more conservative evaluation result.

Considering the influence of impact-induced defect dimensions on material fatigue performance, and further acknowledging the stochastic nature and significant variability inherent in defect depth distributions, a fatigue strength assessment model for damaged axles is developed by integrating defect depth and crack length parameters. Through substitution of Eq. (10) into Eq. (14), Eq. (16) is derived to establish a three-dimensional (3D) full-scale axle fatigue limit model incorporating impact-induced defects.

16 $$\begin{gathered} \Delta \sigma_{{{\mathrm{th}}}} = \frac{{\Delta K_{{{\mathrm{th}}}} }}{{F_{W} \sqrt {\pi \left( {a \times 10^{ - 6} + \frac{1}{\pi }\left( {\frac{{\Delta K_{{{\mathrm{th}}}} }}{{F_{W} \times (2 \times (318 + 153e^{{\left( { - d/64} \right)}} ))}}} \right)^{2} } \right)} }} \hfill \\ d \le 210 \hfill \\ \end{gathered}$$

**Fig. 13:**
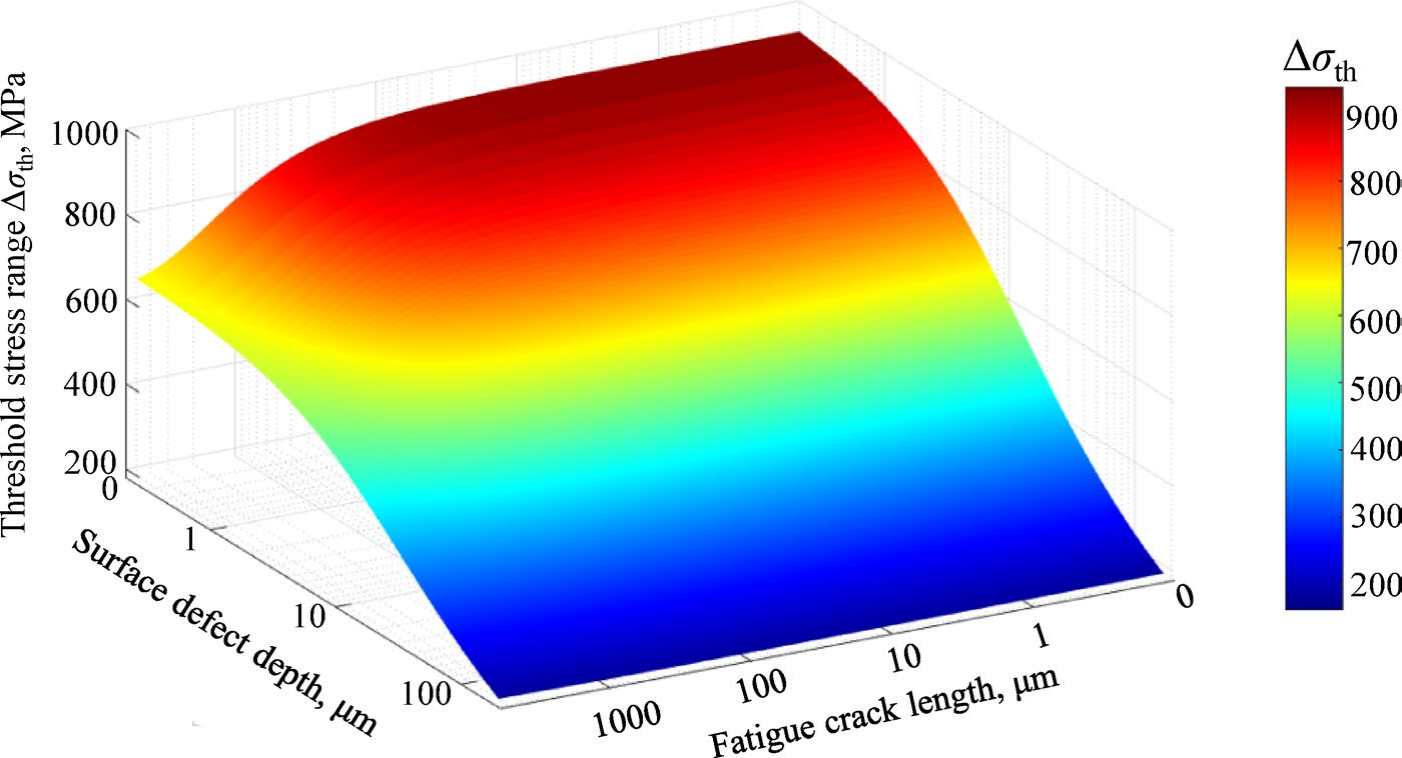
The 3D multivariable evaluation model for fatigue property assessment in full-scale axles containing impact-induced defects.

As demonstrated in Fig. 13, a K-T diagram is constructed by integrating defect depth and crack length. This approach explicitly accounts for the disparities in geometric dimensions, surface quality, loading conditions, and other factors between standardized test specimens and actual axles, as well as for the detrimental influence of surface damage on the material´s fatigue resistance. For railway axles exhibiting varying defect depths and crack lengths, this multivariable model enables the determination of corresponding fatigue limits. This capability facilitates rapid quantitative safety assessments of axles with differing damage severities, thereby providing critical insights for optimizing maintenance strategies targeting high-speed train axles affected by foreign object impact-induced defects.

Using relevant theoretical parameters, the fatigue performance characteristics of full-scale axles are extrapolated from small specimens, which offers certain theoretical guidance for engineering practice. Nevertheless, inherent performance differences exist between actual axles and small test specimens in real applications. Moreover, the operational environment of axles is considerably complex. Therefore, future research will incorporate the size effect to investigate the service performance assessment of full-scale damaged axles under complex environmental conditions.

## Conclusions

This paper focuses on the fatigue performance of surface-strengthened damaged EA4T axle steel. Firstly, axle specimens with impact defects were manufactured, and fatigue tests were carried out to fit the *P-S-N* curve. Stress analysis was performed on the defective areas by integrating finite element simulation results. Finally, based on the El-Haddad formula, a multivariate fatigue performance evaluation model for EA4T full-scale axle steel containing impact defects was established, and the following conclusions were drawn:

1. The residual tensile stress field and stress concentration phenomenon induced by impact defects exerted a significant promoting effect on crack nucleation, serving as critical driving factors for the initiation of fatigue cracking in the vicinity of defect sites. These impact-induced defects lowered the threshold for crack initiation to accelerate the onset of fatigue damage in the material.
2. Fatigue strength in impacted specimens decreases progressively with increasing defect depth. The exponential relationship between full-scale axle fatigue strength and defect size indicates that deeper defects have a more pronounced detrimental effect on fatigue performance.
3. By integrating defect depth and crack length as critical variables, the employment of a multivariate fatigue limit prediction model enables rapid assessment of the fatigue performance of defective axles. Due to the consideration of numerous extreme operational scenarios, the proposed model yields more conservative and reliable safe zone for fatigue loading.

## Supplementary Information

Below is the link to the electronic supplementary material.

Supplementary Material 1

Supplementary Material 2

Supplementary Material 3

## Data Availability

The datasets used during the current study available from the corresponding author on reasonable request. All data generated or analysed during this study are included in this published article and its supplementary information files.
